# A Simple Three-Dimensional Compartmentalized Co-Culture Model for Basal Forebrain and Hippocampal Neurons

**DOI:** 10.3390/biology14091238

**Published:** 2025-09-10

**Authors:** Xiaoman Luo, Jing Li, Zhiyu Deng, Yali Xu, Xixi Li, Miao Ren, Xiangning Li

**Affiliations:** 1 Key Laboratory of Biomedical Engineering of Hainan Province, School of Biomedical Engineering, Hainan University, Sanya 572024, China; luoxiaoman@hainanu.edu.cn (X.L.); lijing-_-@hainanu.edu.cn (J.L.); dengzhiyu@hainanu.edu.cn (Z.D.); xuyali@hainanu.edu.cn (Y.X.); lixixi@hainanu.edu.cn (X.L.); 2 HUST-Suzhou Institute for Brainsmatics, Jiangsu Industrial Technology Research Institute, Suzhou 215123, China

**Keywords:** three-dimensional co-culture, basal forebrain, hippocampus, primary culture

## Abstract

The basal forebrain (BF)-hippocampus (HPC) circuit is indispensable for learning and memory, and in vitro models are essential for dissecting its age-related decline. Nonetheless, current culture methods endure brief survival or confine cells to two dimensions, leaving the circuit’s progressive degeneration refractory to long-term investigation. Here, we developed a simple, three-dimensional (3D) compartmentalized co-culture model that mimics the anatomical organization of BF and HPC neurons. Results demonstrate that basal forebrain cholinergic neurons (BFCNs) co-cultured with primary HPC neurons remain viable for more than two months without exogenous growth factors, significantly promoting BFCNs growth, polarity development, and functional maturation. In this system, BFCNs somata were confined within the hydrogel, whereas cholinergic axons extended toward adjacent hippocampal area, reaching 1681.9 ± 351.8 μm by week 5—significantly longer than in BFCNs monocultures. This model can successfully recapitulate age-dependent progressive neuronal degeneration during long-term culture, validating this long-term co-culture as a platform for studying circuit aging and degeneration. Therefore, this low-cost and highly physiological platform provides a new avenue for in-depth investigations into the mechanisms of neurodegenerative diseases.

## Simple Summary

The interaction between the basal forebrain (BF) and the hippocampus (HPC) regulates memory, learning, and cognitive functions that progressively decline in aging and neurodegenerative conditions. It has been a significant challenge to develop laboratory models for aging studies that could be maintained long enough to observe aging-associated changes. We developed a new, simple, and inexpensive three-dimensional compartmentalized co-culture model for BF and HPC neurons. In this system, by simulating the brain microenvironment, we were able to observe physiological processes of basal forebrain neurons like their development and degeneration. This new model provides a powerful new platform for studying the underlying mechanisms of neurodegenerative diseases.

## 1. Introduction

The interaction between the basal forebrain (BF) and the hippocampus (HPC) regulates memory, learning, and cognitive functions that progressively decline in aging and neurodegenerative conditions [1,2]. Although in vivo studies using techniques like magnetic resonance imaging and positron emission tomography have advanced our understanding of brain structure, neural fiber integrity, aging, neurodegeneration, and plasticity, the complexity of the brain and ethical constraints restrict applications of these knowledge in exploring molecular mechanisms [3,4,5]. Consequently, the development of in vitro models that can precisely recapitulate the BF-HPC interaction is crucial for elucidating the molecular mechanisms of aging and diseases.

However, establishing an ideal in vitro model for the vulnerable basal forebrain cholinergic neurons (BFCNs) presents considerable challenges. Indeed, organotypic slice studies over two decades ago revealed that the hippocampus provides critical trophic support to BFCNs [6,7,8,9], yet this principle has not been fully leveraged in contemporary in vitro models. Conventional primary BFCNs culture techniques still rely on exogenous neurotrophic factors, such as nerve growth factor (NGF) [9,10]. This conventional approach both the cost and complexity of experiments. More critically, its lack of standardized, continuous stimulation fails to mimic the dynamic, target-derived neurotrophic support found in vivo. As a consequence, this method may also mask the true pathological process of diminished endogenous trophic support associated with aging or disease. Secondly, existing models commonly exhibit premature senescence, with strong positive staining for senescence-associated β-galactosidase (SA-β-gal) detectable as early as 18–24 days in culture, which precludes long-term observation of chronic, progressive neuronal pathologies [11,12]. Therefore, exploring new strategies that can overcome the dependence on exogenous factors and support long-term culture is essential to the field.

Recent advances in in vitro modeling have provided a diverse array of platforms for neuroscience research [13,14]. For instance, technologies such as brain organoids [15,16], microfluidic chips [17,18], and 3D printing [19,20] have shown immense potential in simulating brain region architecture and neural circuits. However, these cutting-edge technologies are often associated with complex fabrication processes, high technical barriers, and substantial costs, limiting their widespread application. In contrast, hydrogel-based 3D culture technology, with its operational simplicity, cost-effectiveness, and ability to effectively mimic the mechanical and chemical microenvironment of brain tissue [21] using naturally derived matrices like collagen I [22,23] and Matrigel [17,24], offers an ideal foundational platform for this study.

To address the need for long-term, three-dimensional in vitro modeling of the BF-HPC circuit, we developed a hydrogel-based co-culture system that supports primary 3D culture of both hippocampal and basal forebrain neurons. Using an exogenous factor-free protocol, we achieved extensive neurite outgrowth between segregated neuronal populations. This configuration sustains robust neuronal viability for two months, facilitating continuous monitoring of axonal extension and synaptic maturation recapitulating age-dependent circuit degeneration and thus providing a physiologically relevant and experimentally tractable platform for investigating neural circuits interactions throughout development, aging, and disease.

## 2. Materials and Methods

### 2.1. Acquisition of Hippocampal and Basal Forebrain Cell Suspensions

Primary HPC and BF neurons were obtained from 5–8 P0–P2 neonatal mice using established dissection and dissociation protocols [10,11,12,25]. Neonatal pups were anesthetized on ice, surface sanitized with 75% ethanol, and transferred to a sterile workstation. Following rapid decapitation, the skull was opened along the sagittal suture, and the brain was extracted and placed in precooled calcium- and magnesium-free Hank’s Balanced Salt Solution (HBSS; Gibco, Grand Island, NY, USA). Under a stereomicroscope (PXS6555T-B5; Shanghai Cewei Photoelectric Technology Co., Ltd., Shanghai, China), the BF and HPC were isolated, and meninges and blood clots were removed. The tissues were minced to <1 mm^3^, centrifuged to remove HBSS, and digested with 0.25% Trypsin-EDTA (Gibco, Grand Island, NY, USA) at 37 °C for 10 min. After trypsin was removed, HBSS with Ca^2+^/Mg^2+^ (Gibco, Grand Island, NY, USA) and 8% (*v*/*v*) DNase I (Roche, Basel, Switzerland) was added to the tissue, followed by a 10-min incubation. After pipetting, the cell suspension was filtered through a 40 µm cell strainer (Corning, Corning, NY, USA), centrifuged, and resuspended in neuronal culture medium (Neurobasal, Gibco, Grand Island, NY, USA; supplemented with 2% B27, Gibco, Grand Island, NY, USA; and 0.5 mM GlutaMAX, Gibco, Grand Island, NY, USA). Viable cell density was determined using a hemocytometer and trypan blue staining and adjusted to 5.0 × 10^4^ cells/µL, with a total of 1.0 × 10^5^ cells/well per brain region used for model construction.

Animals used in this study were SPF-grade C57BL/6 mice bred and housed by Guangzhou Ruige Biotechnology Co., Ltd. (Guangzhou, China). Animal experiments were conducted in accordance with the guidelines of the Animal Ethics Committee of Hainan University.

### 2.2. Model Construction

**Figure 1:**
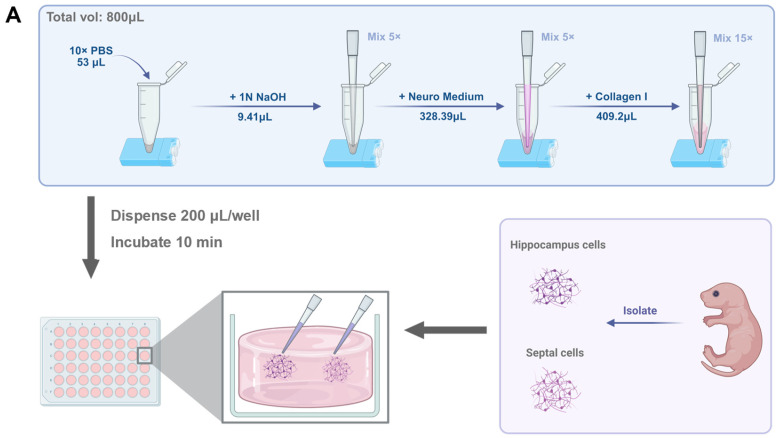

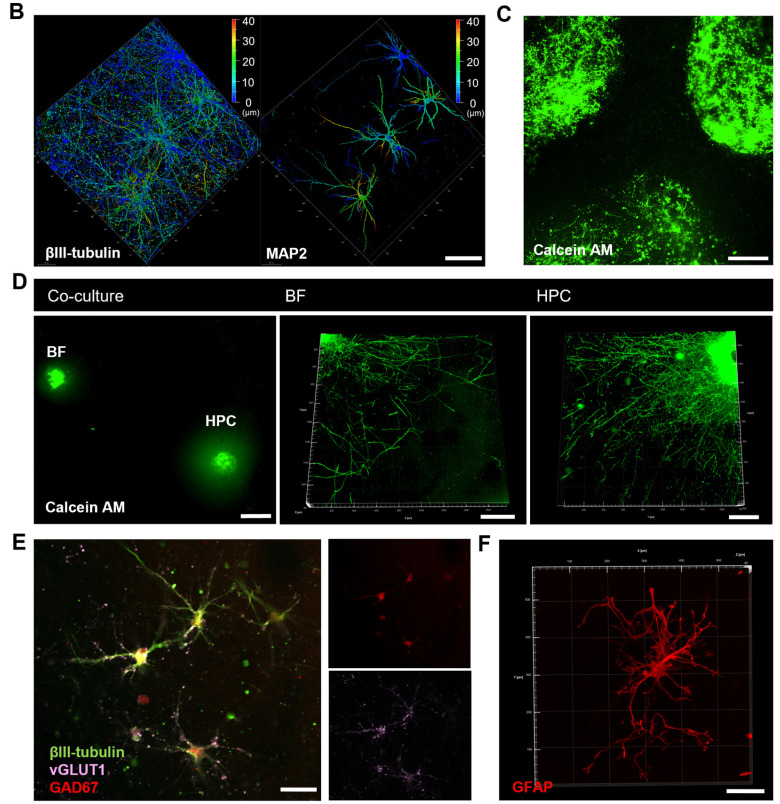
Construction and characterization of the compartmentalized basal forebrain (BF) and hippocampal (HPC) co-culture system. (**A**) Schematic workflow illustrating the seeding of BF and HPC neurons into a semi-cured Collagen I hydrogel to establish spatially distinct populations. (**B**) A 3D reconstruction of primary hippocampal neurons at 22 days in vitro (DIV) within the Collagen I hydrogel, stained for neuronal markers βIII-tubulin (Left) and MAP2 (Right), where colors represent Z-depth as indicated by the color bar. Z-depth: 40 μm; scale bar: 100 μm. (**C**) Live-cell imaging with Calcein-AM at DIV 7 showing three distinct seeding zones of primary hippocampal cells. Scale bar: 500 μm. (**D**) Representative images at DIV 14 depicting the spatial separation of co-cultured BF and HPC neurons. Left: Low-magnification overview of the two compartments. Scale bar: 1000 μm. Middle and Right: Local 3D reconstructions of the BF and HPC neurite fields, respectively. Z-depths = 133 μm and 313 μm. Scale bars: 300 μm. (**E**) Multiplex immunostaining at DIV 30 showing excitatory (VGLUT1, magenta) and inhibitory (GAD67, red) neurons, co-labeled with neuronal marker βIII-tubulin (green). Scale bar: 50 μm. (**F**) Representative astrocyte morphology at DIV 30, visualized by immunostaining for GFAP (red). Scale bar: 100 μm.

Collagen I hydrogel was selected as the matrix to simulate the microenvironment of neural tissue due to its excellent performance in cell distribution uniformity, culturing duration, and immunofluorescence compatibility. Collagen I hydrogel was prepared by mixing 53 µL 10× PBS (Gibco, Grand Island, NY, USA), 9.41 µL 1N NaOH (Merck, Darmstadt, Germany), 328.39 µL neuronal culture medium and 409.253 µL Collagen I stock solution (Corning, Corning, NY, USA) (Figure 1A). The pH was adjusted to 7.2–7.4, and the final collagen concentration was 2.0 mg/mL. The hydrogel precursor solution was aliquoted at 200 µL per well into 48-well plates (NEST Biotechnology, Wuxi, China) and allowed to rest at room temperature for 10 min to reach a semi-cured state, characterized by retained fluidity during initial gelation. Subsequently, 2 µL of primary HPC and BF neuronal suspensions were carefully pipetted onto opposite edges of the semi-cured hydrogel, respectively. The hydrogel’s viscosity two distinct cell compartments were established and maintained (Figure 1B). Complete gelation was achieved by incubating the culture plates at 37 °C for 30 min in an incubator (Heracell VIOS 160i LK, Thermo Fisher, Waltham, MA, USA), after which culture medium was added to the top. Half of the medium was replaced weekly.

### 2.3. Live-Cell Imaging

The morphology of living neurons in the 3D culture was visualized using Calcein-AM (Thermo Fisher, Waltham, MA, USA) staining. Cells were incubated for 15–20 min in an incubator (Heracell VIOS 160i LK, Thermo Fisher, Waltham, MA, USA) with culture medium containing 0.08% (*v*/*v*) Calcein-AM. Fluorescence was observed under a Nikon ECLIPSE Ti2-E confocal microscope (Nikon, Tokyo, Japan; 488 nm channel) using NIS-Elements AR software (Version 6.0) to capture images. Three-dimensional reconstruction of the imaging data was performed using the 3D Viewer in NIS-Elements AR software.

### 2.4. Immunofluorescence Staining

Immunostaining was performed to detect specific proteins. Cells were fixed with 4% PFA (Sigma, St. Louis, MO, USA) for 30 min, followed by three rinses with PBS (Sigma, St. Louis, MO, USA). Permeabilization and blocking were conducted using 5% BSA (Sigma, St. Louis, MO, USA) and 0.3% Triton X-100 (Sigma, St. Louis, MO, USA) for 2 h, followed by three PBS rinses. The samples were incubated with primary antibodies for 24 h at 4 °C, followed by three PBS rinses. Then, the samples were incubated with secondary antibodies for 2 h at 37 °C in the dark, followed by three PBS rinses. Nuclei were counterstained with DAPI (Thermo Fisher, Waltham, MA, USA) for 10 min in the dark, followed by three PBS rinses. Imaging was performed using a confocal microscope.

Primary antibodies included: Chicken anti-MAP2 (1:1000, Abcam, Cambridge, UK; Cat. No. ab5392), Mouse anti-βIII tubulin (1:500, Abcam, Cambridge, UK; Cat. No. ab78078), Rabbit anti-Synapsin I (1:500, Merck, Darmstadt, Germany; Cat. No. AB1543P), Goat anti-ChAT (1:800, Sigma, St. Louis, MO, USA; Cat. No. AB144P), Rabbit anti-VAChT (1:500, Abcam, Cambridge, UK; Cat. No. AB235201), Rabbit anti-GFAP (1:1000, Dako, Glostrup, Denmark; Cat. No. Z0334), Rabbit anti-vGlut1 (1:500, Abcam, Cambridge, UK; Cat. No. AB272913), Chicken anti-GAD67 (1:500, Abcam, Cambridge, UK; Cat. No. AB75712). Secondary antibodies included: Donkey anti-rabbit IgG Alexa Fluor 488 (1:500, Thermo Fisher, Waltham, MA, USA; Cat. No. A21206), Donkey anti-mouse IgG Alexa Fluor 488 (1:500, Thermo Fisher, Waltham, MA, USA; Cat. No. A21202), Donkey anti-rabbit IgG Alexa Fluor 594 (1:500, Thermo Fisher, Waltham, MA, USA; Cat. No. A21207), Donkey anti-mouse IgG Alexa Fluor 594 (1:500, Thermo Fisher, Waltham, MA, USA; Cat. No. A21203), Donkey anti-goat IgG Alexa Fluor 647 (1:500, Thermo Fisher, Waltham, MA, USA; Cat. No. A21447), Donkey anti-mouse IgG Alexa Fluor 405 (1:500, Thermo Fisher, Waltham, MA, USA; Cat. No. A48257), Donkey anti-rabbit IgG Alexa Fluor 405 (1:500, Thermo Fisher, Waltham, MA, USA; Cat. No.A48258).

### 2.5. Image Processing and Quantification

Images were processed using Fiji/ImageJ software (1.54p, National Institutes of Health, Bethesda, MD, USA). Regions of interest (ROIs) corresponding to neuronal somata and dendrites were defined using the MAP2 channel. Maximum intensity projections (MIPs) of MAP2 were converted to 8-bit grayscale and subjected to automatic thresholding (Mean method, dark background) to generate binary masks. Bright outliers (radius < 2 pixels, intensity > 50) were removed to minimize noise. The Analyze Particles tool identified ROIs by selecting particles with area ≥ 35 square pixels (equivalent to ≥11.54 μm^2^ at 0.5741734 μm/pixel resolution) and circularity 0.00–1.00, which were added to the ROI Manager.

MAP2-derived ROIs were applied to MIPs of VAChT, ChAT, and p21 channels to quantify mean fluorescence intensity. Background intensity was calculated as the average of five randomly selected unstained regions per image. To correct for nonspecific signals, values were normalized using the formula: $R=\frac{Mean\;-\;{Mean}_{bg}}{\;{Mean}_{bg}}$ , where *R* is the normalized relative fluorescence intensity, *Mean* is the mean fluorescence intensity and *Mean_bg_* is the background intensity. For nuclear p21 analysis, DAPI channel MIPs were processed similarly to MAP2 to identify nuclei and quantify p21 expression within them.

To quantify SYN1 puncta density on MAP2-positive dendrites, the ImageJ plugin Simple Neurite Tracer (https://imagej.net/plugins/snt/index, accessed on 1 September 2025) was used to trace and measure the number and length of primary and secondary dendritic branches. SYN1 puncta were manually counted, and density was calculated as the number of SYN1-positive puncta divided by the total traced MAP2 dendritic length.

All quantifications were performed on at least three independent biological replicates to ensure reproducibility. Data were exported as CSV files for statistical analysis.

### 2.6. Analysis of Neurite Outgrowth and Complexity

To monitor neurite outgrowth over a two-month period in a hydrogel co-culture system, live-cell imaging was performed at weeks 1, 3, 5, 7, and 9. Due to the dense and outwardly extending nature of neurites, which complicates tracing individual paths, neurite length was measured as the straight-line distance from the soma edge to the neurite tip using Fiji/ImageJ software (1.54p, National Institutes of Health, Bethesda, MD, USA). At each time point, a minimum of 24 neurons were analyzed, sourced from 4 hydrogel culture units across 3 independent experiments.

To characterize morphological complexity at the developmental peak (week 5, corresponding to maximal neurite length), Sholl analysis was conducted on three representative samples from each of the BF and HPC regions. Concentric circles were drawn from the manually selected soma center, with the initial circle enclosing the soma and subsequent circles spaced at 400 µm intervals to cover the typical neurite extension range. Neurite density was quantified by measuring the average fluorescence intensity within each concentric ring, followed by background correction using the mean intensity from three randomly selected background regions and numerical normalization. All data were plotted and statistically analyzed using GraphPad Prism 9.5 software (GraphPad Software, San Diego, CA, USA).

### 2.7. Calcium Imaging

Calcium imaging was performed to evaluate the functional activity of the neural networks. Cultures were first washed with calcium- and magnesium-free HBSS (Gibco, Grand Island, NY, USA). Subsequently, Cultures were loaded with 1 µM Fluo-4 AM (Thermo Fisher, Waltham, MA, USA) in serum-free medium and incubated for 20 min in an incubator (Heracell VIOS 160i LK, Thermo Fisher, Waltham, MA, USA). After loading Fluo-4 AM, the solution was replaced with calcium- and magnesium-free HBSS (Gibco, Grand Island, NY, USA). Images were acquired using a Nikon ECLIPSE Ti2-E confocal microscope with NIS-Elements AR software (Nikon, Tokyo, Japan) at 200 ms intervals for a duration of 1 min (resolution: 512 × 512, 10× objective, 5× zoom). Fiji/ImageJ software (1.54p, National Institutes of Health, Bethesda, MD, USA) was used for subsequent analysis. All cells exhibiting a response within the field of view were defined as regions of interest (ROIs). Fluorescence signals from regions of interest (ROIs) were normalized as $∆F/F=\frac{F{\text{-}F}_{\mathrm{bg}}}{F_{\mathrm{bg}}}$ , where *F* is the instantaneous fluorescence intensity and *F_bg_* is the baseline fluorescence. Changes in ∆*F*/*F* over time were plotted using GraphPad Prism 9.5 software (GraphPad Software, San Diego, CA, USA).

### 2.8. Data Analysis

All data were analyzed using GraphPad Prism 9.5 software (GraphPad Software, San Diego, CA, USA). Each experimental group consisted of at least three independent biological replicates. Results were expressed as mean ± standard deviation (SD). The Shapiro-Wilk test was used to assess the normality of distribution. Changes in neurite growth and P21 expression were assessed by one-way or two-way analysis of variance (ANOVA), followed by Tukey’s test for multiple comparisons. Comparisons of dendritic length and branch number, cholinergic marker expression, and SYN1 density between monoculture and co-culture groups were performed using an unpaired, two-tailed Student’s *t*-test, assuming equal variances (confirmed by a non-significant F-test with *p* > 0.05). Statistical significance was defined as *p* < 0.05.

## 3. Results

### 3.1. Characterization of the Compartmentalized Co-Culture System

We established a 3D co-culture system based on a Collagen I hydrogel that enables the spatial separation of BF and HPC neuronal cell bodies, providing distinct regions for neurite observation (Figure 1A). In this system, primary hippocampal neurons exhibited rich dendritic branching and formed a dense neurite network (Figure 1B). The fabrication method successfully created two to three independent cell seeding zones. To minimize inter-regional neurite overlap and enhance observation clarity, these zones were spaced 4–6 mm apart, which effectively reduced the intermingling of neurites (Figure 1C).

At 14 days in vitro (DIV), local 3D reconstructions revealed robust neurite extension, with BF and HPC neurites projecting horizontally outward from their respective somatic zones (Figure 1D, middle and right panels). By DIV 30, multiplex immunostaining for βIII-tubulin, VGLUT1, and GAD67 identified both excitatory and inhibitory neurons, including a subpopulation that co-expressed VGLUT1 and GAD67 (Figure 1E). Furthermore, GFAP immunostaining revealed astrocytes with complex, branched morphologies within the culture (Figure 1F). Collectively, these observations confirm the capacity of this system to maintain diverse neural populations and support their structural integration.

### 3.2. Co-Culturing Promotes Polarized Development of Basal Forebrain Neurons

**Figure 2:**
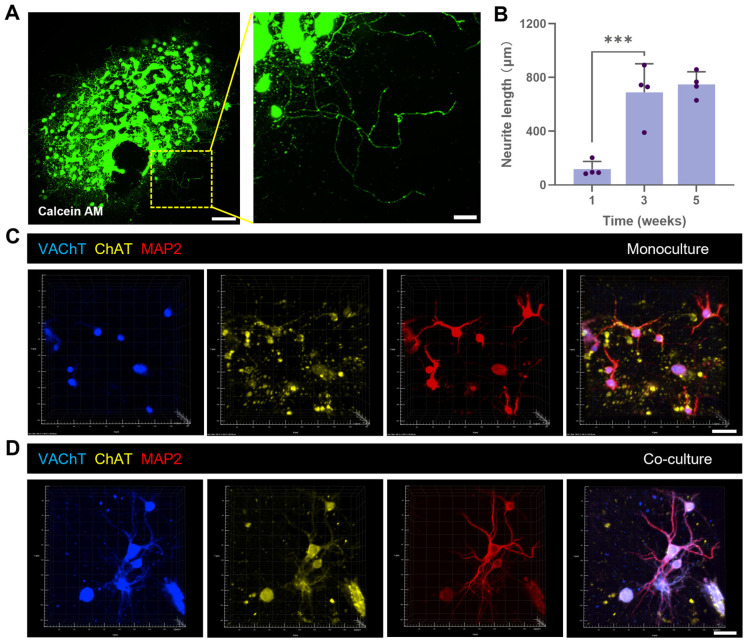

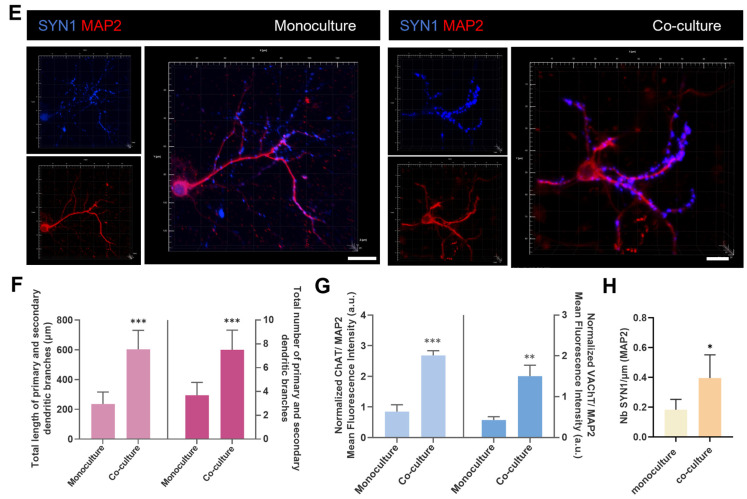
Co-culturing promotes polarity, maturation, and synaptic development of basal forebrain (BF) neurons. (**A**) Live-cell imaging of BF neurons under monoculture conditions at week 1, showing simple morphology. Maximum intensity projection, Z = 154 μm. Scale bar: 200 μm; inset: 50 μm. (**B**) Quantification of neurite length in BF neurons under monoculture conditions at 1, 3, and 5 weeks, with each dot representing an individual hydrogel culture unit. (**C**,**D**) Representative fluorescence images of BF neurons at 14 days in vitro (DIV) under (**C**) monoculture and (**D**) co-culture conditions. Stains: vesicular acetylcholine transporter (VAChT; blue), choline acetyltransferase (ChAT; yellow), and microtubule-associated protein 2 (MAP2; red). Scale bar: 30 μm. (**E**) Immunostaining for Synapsin I (SYN1) in BF neurons at DIV 14 under monoculture (left) and co-culture (right) conditions. Stains: SYN1 (blue), MAP2 (red). Scale bar: 20 μm. (**F**) Quantification of the total length of primary and secondary dendrites and the total number of dendritic branches per BF neuron at DIV 14. (**G**) Quantification of the mean fluorescence intensity of ChAT and VAChT in BFCNs at DIV 14. (**H**) Quantification of SYN1 puncta density along MAP2-positive dendrites of BF neurons at DIV 14. * *p* < 0.05, ** *p* < 0.01, *** *p* < 0.001. Data were presented as mean ± standard deviation (SD).

The co-culture system significantly enhanced polarized development and maturation of BF neurons. Under monoculture conditions, BF neurons exhibited simple branching, shorter neurite lengths, and an atypical morphology (Figure 2A). The average neurite length was 119.0 ± 56.4 μm at week 1, reaching 689.9 ± 212.4 μm by week 3 and 748.2 ± 94.4 μm by week 5 (Figure 2B). Within this population, the cholinergic markers VAChT and ChAT were predominantly localized to the soma and proximal neurites of marker-positive cells (Figure 2C).

In stark contrast, BF neurons in the co-culture system developed complex dendritic arbors at DIV 14. The total length of primary and secondary dendrites increased from 236.3 ± 80.0 μm in monoculture to 603.4 ± 127.4 μm in co-culture, accompanied by a significant increase in branch number (Figure 2E,F). Furthermore, in the cholinergic subpopulation, the expression of VAChT and ChAT was distributed throughout the entire neurite arbor (Figure 2D), with quantification confirming a significant increase in their fluorescence intensity (Figure 2G). As an indicator of presynaptic maturation, the density of Synapsin I (SYN1) puncta along neurites increased from 0.2 ± 0.1 puncta/μm in monoculture to 0.4 ± 0.2 puncta/μm in co-culture (Figure 2E,H). Together, these enhanced structural and molecular features indicated improved maturation of BF neurons, particularly the cholinergic population, under co-culture conditions.

### 3.3. Dynamics of Neuronal Maturation and Degeneration in Long-Term Co-Culture

**Figure 3:**
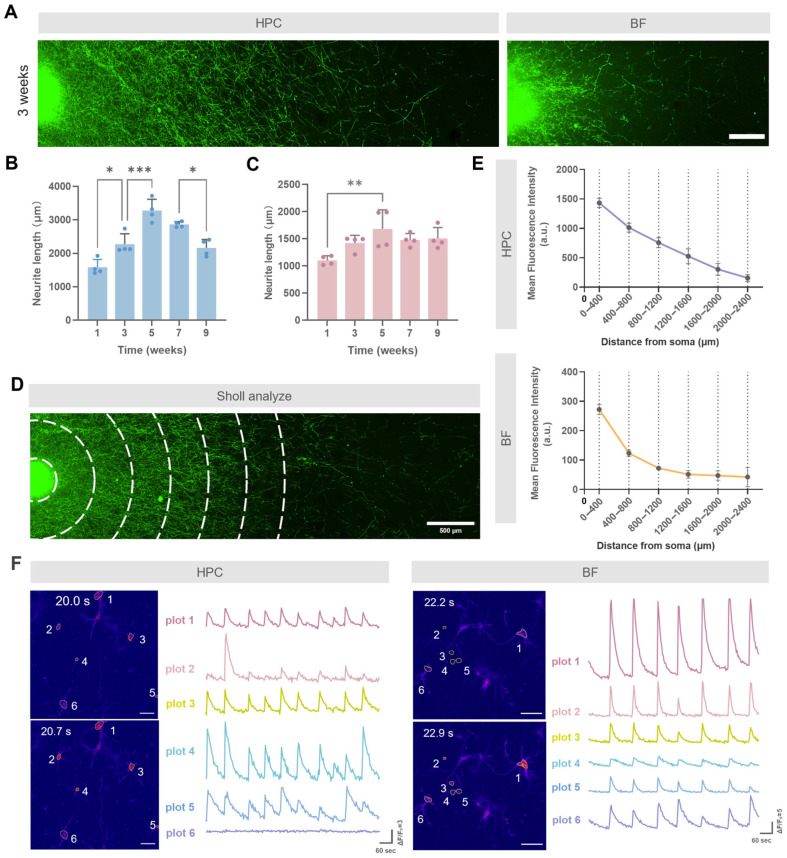
Neurite growth dynamics and functional network activity in the co-culture system. (**A**) Representative images of neurite outgrowth from hippocampal (HPC) and basal forebrain (BF) neurons at 3 weeks. Cells were seeded at a density of 1.0 × 10^5^ cells/mL. Scale bar: 300 μm. (**B**,**C**) Quantification of (**B**) HPC and (**C**) BF neurite length over 9 weeks. Data were presented as mean ± standard deviation (SD), with each dot representing an individual hydrogel culture unit (n = 4). (**D**) Representative fluorescence image showing the setup for Sholl analysis, with concentric circles centered on the somatic region to assess neurite density distribution. Scale bar: 500 μm. (**E**) Neurite density profiles showing the average fluorescence intensity as a function of distance from the soma for HPC (**top**) and BF (**bottom**) neurons. (**F**) Spontaneous Ca^2+^ oscillations in HPC and BF regions at 38 days in vitro. Pseudocolor ‘fire’ mode shows fluorescence intensity at selected time points, where warmer colors (e.g., yellow and red) indicate higher Ca^2+^ concentration. Traces for regions of interest (ROIs) 1−6 show ΔF/F_0_ over 60 s. Scale bar: 50 μm. Statistical significance in (**B**,**C**) was determined by one-way ANOVA with Tukey’s post-hoc test. * *p* < 0.05, ** *p* < 0.01, *** *p* < 0.001.

To quantify neurite outgrowth dynamics in the compartmentalized co-culture system, we evaluated neurite lengths in both HPC and BF neurons at multiple time points. One-way ANOVA revealed a significant time effect for both populations (HPC: *F* (4,15) = 25.63, *p* < 0.0001; BF: *F* (4,15) = 4.352, *p* = 0.0156). HPC neurite lengths increased from 1585.7 ± 232.6 μm at week 1 to a peak of 3280.5 ± 339.0 μm at week 5, then declined to 2864.3 ± 85.7 μm at week 7 and 2164.1 ± 250.5 μm at week 9 (Figure 3A,B). BF neurite lengths rose from 1102.5 ± 85.3 μm at week 1 to a peak of 1681.9 ± 351.8 μm at week 5, stabilizing at 1478.7 ± 117.9 μm at week 7 and 1506.7 ± 201.0 μm at week 9 (Figure 3C). Sholl analysis at week 5 confirmed highest neurite density near the soma, decreasing distally (Figure 3E). Calcium imaging at DIV 38 showed spontaneous intracellular Ca^2+^ oscillations (Figure 3F). Neurons in the HPC region exhibited asynchronous oscillation patterns, whereas BF neurons displayed distinct and highly synchronized oscillations.

**Figure 4:**
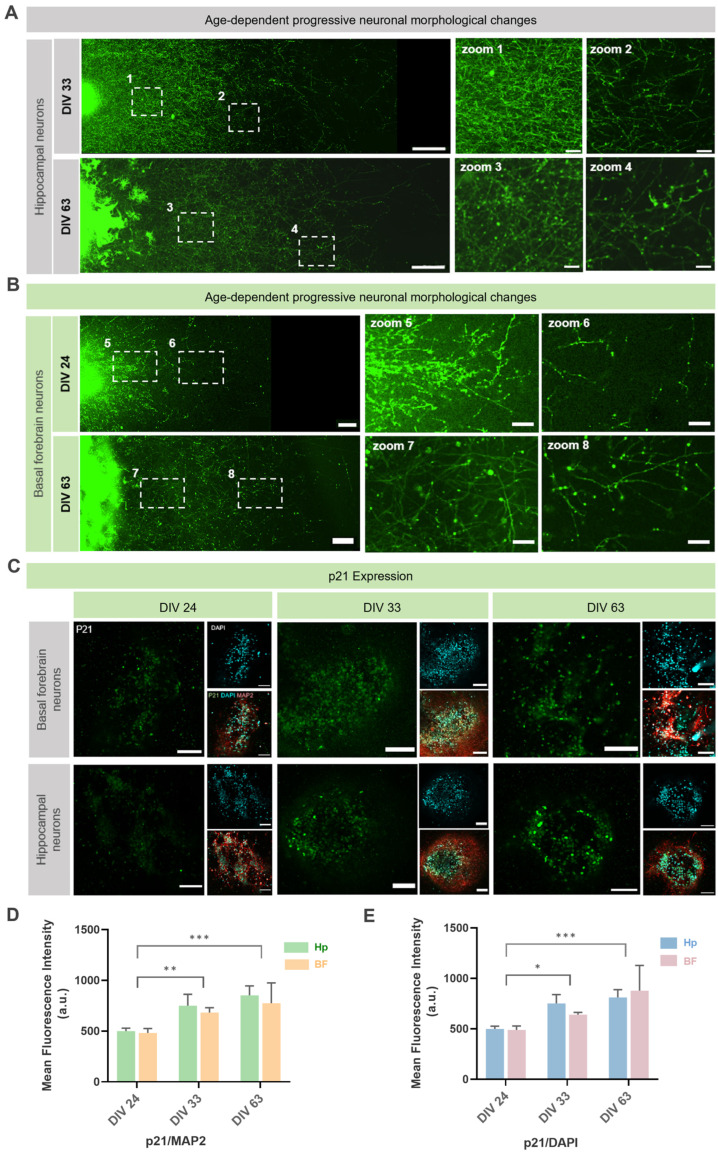
Degenerative morphological and molecular changes in long-term co-culture. (**A**) Live-cell imaging of hippocampal (HPC) neurons at 33 and 63 days in vitro (DIV), depicting proximal and distal neurite morphology. Insets show magnified views of degenerative features like swellings and tortuosity. Scale bar: 500 μm; inset: 50 μm. (**B**) Basal forebrain (BF) neurons at DIV 24 and DIV 63, illustrating progressive changes in neurite morphology. Insets show magnified views. Scale bar: 200 μm; inset: 50 μm. (**C**) Immunofluorescence staining for the senescence-associated marker p21 (green), the neuronal marker microtubule-associated protein 2 (MAP2; red), and nuclei (DAPI; blue) in BF and HPC neurons across different culturing durations. Scale bar: 100 μm. (**D**,**E**) Quantification of the mean fluorescence intensity for p21 co-localized with (**D**) MAP2 and (**E**) DAPI. Data were presented as mean ± standard deviation (SD), n = 24. Statistical significance was determined by two-way ANOVA with Tukey’s post-hoc test. * *p* < 0.05, ** *p* < 0.01, *** *p* < 0.001.

To characterize progressive degeneration, we conducted longitudinal imaging at weeks 1, 3, 5, 7, and 9 in our co-culture systems. Based on preliminary observations of early morphological degenerative signs in BF neurons compared to HPC neurons, we selected representative time points to highlight region-specific vulnerability. Four independent experiments consistently showed distal axonal swellings in BF neurons around week 3 (~DIV 24), accompanied by proximal swelling, while HPC neurons exhibited similar distal changes after week 4 (~DIV 33), with proximal neurites initially stable. By DIV 63, both HPC and BF neurons displayed evident degeneration, including proximal swellings in HPC neurons. Thus, we imaged at DIV 24, 33, and 63 to capture this temporal and regional progression (Figure 4A,B), supporting the model’s utility in studying age-related neurodegenerative processes.

At the molecular level, we investigated changes in the senescence-associated marker p21. Analysis of p21 expression across all cell nuclei (normalized to DAPI) revealed a significant increase over time (*F* (2,12) = 14.15, *p* = 0.0007; Figure 4D). To determine if this trend was specifically attributable to the neuronal population, we next quantified p21 expression selectively within MAP2-positive neurons. This neuron-specific analysis confirmed a significant increase in p21 levels as well (*F* (2,12) = 15.00, *p* = 0.0005; Figure 4E). No significant differences in these expression patterns were observed between BF and HPC regions (p21/DAPI: *F* (1,12) = 0.1136, *p* = 0.7419; p21/MAP2: *F* (1,12) = 1.209, *p* = 0.2931), indicating a consistent response across both populations (Figure 4D,E). These findings demonstrate the utility of this system for tracking both developmental trajectory and progressive degenerative alterations in long-term neural cultures.

## 4. Discussion

In this study, we established and validated a simple yet robust 3D-compartmentalized co-culture model that simulates the interaction between BF and HPC neurons and supports culturing for over two months. The core innovation lies in recapitulating the endogenous, HPC-derived trophic support for BFCNs, thereby obviating the reliance on exogenous growth factors common in conventional models. This is critical, as the addition of exogenous growth factors not only increases costs but may mask intrinsic pathological processes, such as the age-related declines central to neurodegenerative diseases [26]. This design preserves the entire endogenous neurotrophin metabolic pathway, particularly the conversion of proNGF to its mature form, mNGF—a key process known to be dysregulated in Alzheimer’s disease (AD) and other neurodegenerative conditions [27]. Therefore, this model provides a powerful, low-cost, and tractable platform for studying long-term development, senescence, and pathology of neurons under more physiologically relevant conditions.

The physiological relevance and success of the model are directly demonstrated by its potent promotion of BFCNs survival, polarized development, and functional maturation, evidenced by the extensive distribution of VAChT and SYN1 along distal neurites. In contrast, BFCNs under monoculture conditions, lacking HPC support, exhibit severe developmental deficits, a finding that aligns with the principle of target-derived trophic support observed in organotypic studies [6,7,8,9] and highlights the successful in vitro reconstruction of this dependency. Under this physiological support, neurons displayed robust growth comparable to that in advanced microfluidic models [17]. Concurrently, we observed a predominantly horizontal growth pattern of neurons, likely resulting from hydrogel fiber network arrangement induced by hydrostatic pressure during the seeding of cell suspension in semi-cured hydrogels [17,28].

Furthermore, the utility of this model extends beyond simulating a healthy state to encompass the recapitulation of degenerative phenotypes associated with prolonged culture periods. We observed a classic “dying-back” pattern of axonal degeneration, with BFCNs exhibiting degenerative morphological changes earlier than hippocampal neurons. This aligns with the clinical feature of preferential cholinergic system vulnerability observed in the brains of AD patients [29], demonstrating that our model reproduces brain region-specific vulnerability in vitro. This may arise from the higher metabolic demands of BFCNs and their greater sensitivity to oxidative stress, consistent with upregulated metabolic genes in aged rodent BFCNs [30,31,32,33]. Oxidative stress impairs NGF signaling by downregulating the expression and retrograde transport of its high-affinity receptor, TrkA, creating a vicious cycle. This shift favors pro-apoptotic p75NTR pathways, accelerating neurodegeneration [11,31]. Thus, our platform is not merely a culture system but also a disease model capable of endogenously driving and displaying key pathological cascades.

We also found that the expression of p21, a key senescence marker driven by DNA damage responses, increases significantly over time in both regions, correlating with progressive degeneration [34,35]. However, despite the greater morphological vulnerability of BFCNs, their p21 expression levels showed no regional difference, suggesting p21 may be a universal marker of the culture system’s overall cumulative stress, rather than a direct indicator of BFCN-specific vulnerability. The focus of this study is on the model’s construction and the initial validation of its potential. A comprehensive characterization of senescence in this model will require assessing additional markers, such as SA-β-gal activity or p16 expression, in future studies [36].

Despite the demonstrated feasibility of our model, certain limitations exist to guide future improvements. First, the current model lacks precise control over the physical distance between brain regions, limiting quantitative analysis of parameters like synaptic connection probability. Our future work will focus on integrating customized microfluidic molds to regulate neuronal layout and connectivity, enabling dose-dependent studies of trophic support by varying inter-region distances. Second, the lack of an on-stage incubator limits long-term functional monitoring such as calcium imaging. Future studies should integrate such systems to enable extended functional readouts. Additionally, combining multi-electrode array recordings will allow testing hypotheses on network synchrony and bursting activities during degeneration. Integrating real-time acetylcholine biosensors will provide direct assessment of synaptic function, enhancing understanding of BF-HPC circuit function and dysfunction.

On a biological level, future iterations of the model should also aim to capture the full complexity of the degenerative process. For instance, we observed that some neurons co-expressed excitatory and inhibitory markers, potentially reflecting incomplete differentiation or an adaptive response, and their functional impact warrants further exploration. More importantly, recent in vivo evidence from non-human primates suggests that basal forebrain GABAergic neurons may be particularly susceptible to age-related decline [37], indicating that future models should incorporate diverse neuronal subtypes in order to more comprehensively simulate the complex pathology of age-related degeneration.

## 5. Conclusions

This study developed and validated a low-cost, user-friendly, and physiologically relevant 3D co-culture model of the basal forebrain and hippocampus. By leveraging endogenous trophic support, this model successfully simulates the long-term interactions, developmental maturation, and senescence-associated degeneration between BF and HPC neurons. It thus provides a platform to advance our understanding of the mechanisms underlying neurodegenerative diseases.

## Data Availability

All data related to this study are contained within the manuscript. Data can be obtained from the corresponding authors on request.
