# Correction: Luo et al. A Simple Three-Dimensional Compartmentalized Co-Culture Model for Basal Forebrain and Hippocampal Neurons. *Biology* 2025, *14*, 1238

**DOI:** 10.3390/biology14111514

**Published:** 2025-10-29

**Authors:** Xiaoman Luo, Jing Li, Zhiyu Deng, Yali Xu, Xixi Li, Miao Ren, Xiangning Li

**Affiliations:** 1 Key Laboratory of Biomedical Engineering of Hainan Province, School of Biomedical Engineering, Hainan University, Sanya 572024, China; 2 HUST-Suzhou Institute for Brainsmatics, Jiangsu Industrial Technology Research Institute, Suzhou 215123, China

## Text Correction

There was an error in the original publication [1]. During the revision process, a paragraph was inadvertently deleted in Section 3.2. This missing paragraph contains the description of the quantitative data for Figure 2E–H).

A correction has been made to Section 3.2, Co-Culturing Promotes Polarized Development of Basal Forebrain Neurons. The corrected text for this section now reads:

“The co-culture system significantly enhanced polarized development and maturation of BF neurons. Under monoculture conditions, BF neurons exhibited simple branching, shorter neurite lengths, and an atypical morphology (Figure 2A). The average neurite length was 119.0 ± 56.4 μm at week 1, reaching 689.9 ± 212.4 μm by week 3 and 748.2 ± 94.4 μm by week 5 (Figure 2B). Within this population, the cholinergic markers VAChT and ChAT were predominantly localized to the soma and proximal neurites of marker-positive cells (Figure 2C).

In stark contrast, BF neurons in the co-culture system developed complex dendritic arbors at DIV 14. The total length of primary and secondary dendrites increased from 236.3 ± 80.0 μm in monoculture to 603.4 ± 127.4 μm in co-culture, accompanied by a significant increase in branch number (Figure 2E,F). Furthermore, in the cholinergic subpopulation, the expression of VAChT and ChAT was distributed throughout the entire neurite arbor (Figure 2D), with quantification confirming a significant increase in their fluorescence intensity (Figure 2G). As an indicator of presynaptic maturation, the density of Synapsin I (SYN1) puncta along neurites increased from 0.2 ± 0.1 puncta/μm in monoculture to 0.4 ± 0.2 puncta/μm in co-culture (Figure 2E,H). Together, these enhanced structural and molecular features indicated improved maturation of BF neurons, particularly the cholinergic population, under co-culture conditions”.

The authors state that the scientific conclusions are unaffected. This correction was approved by the Academic Editor. The original publication has also been updated.
